# Exploring health needs and the double burden of disease in adults attending public health facilities in the Iraqi Kurdistan

**DOI:** 10.3389/fpubh.2025.1649273

**Published:** 2025-10-23

**Authors:** Stefania Moramarco, Mariagrazia Cicala, Faiq B. Basa, Gorgees S. Akhshirsh, Antonia Bezenchek, Rafal S. L. Adhama, Bayar S. Azeez, Sivar A. Qadir, Shahla O. Salih, Luma H. H. Alhanabadi, Iuri Fanti, Berivan A. Tofiq, Daniele Di Giovanni, Leonardo Emberti Gialloreti, Paola Scarcella

**Affiliations:** ^1^Department of Biomedicine and Prevention, University of Rome Tor Vergata, Rome, Italy; ^2^Rizgary Teaching Hospital, Erbil, Iraq; ^3^Computer Systems Engineering, Erbil, Iraq; ^4^Informa-PRO, Rome, Italy; ^5^Directorate of Health, Sulaimaniya, Iraq; ^6^Department of Statistics and Informatics, University of Sulaimaniya, Sulaimaniya, Iraq; ^7^Primary Health Care Department, Preventive Health Affairs Directorate, Duhok, Iraq; ^8^EuResist Network GEIE, Rome, Italy; ^9^Directorate of Health, Halabja, Iraq; ^10^Department of Human Sciences, LUMSA University, Rome, Italy

**Keywords:** non-communicable diseases, communicable diseases, double burden of disease, epidemiological surveillance, public health records, DHIS2, Iraq

## Abstract

**Objective:**

We analyzed the health needs of the adult population accessing public health facilities in the Iraqi Kurdistan, a region facing both demographic and epidemiological transitions while undergoing post-crisis recovery. We investigates the prevalence and distribution of communicable and non-communicable diseases using real-world data from a digital surveillance system.

**Methods:**

Data collected across public health centers (PHC) were extracted from the KRG-DHIS2 digital health platform. All records from adult patients were coded using the WHO ICD-10. Diagnoses were grouped into: Communicable, maternal, neonatal and nutritional diseases (CMNNDs), Non-communicable diseases (NCDs), Injuries, Ill-defined diseases. Statistical analyses included descriptive statistics, age-related trends and sex-specific comparisons.

**Results:**

A total of 1,040,695 health events were recorded (years 2016–2022) across 96 PHC: 899,173 were classified as either CMNNDs (41.1%) or NCDs (58.9%). Significant sex and age differences were observed across all major diagnostic categories. NCDs were more common in all age groups and increased significantly with age, while CMNNDs predominated among younger adults. Females accounted for 63.4% of all health events and exhibited higher rates of visits for endocrine, genitourinary, and hematologic conditions. Respiratory system diseases were the most common diagnoses across all ages, followed by genitourinary and digestive system diseases.

**Conclusion:**

This study provides critical evidence for understanding health service utilization and disease burden in Iraqi Kurdistan, using a real-time digital surveillance system. The findings confirm the presence of a double burden of disease in a population undergoing rapid transition and underscore the need for integrated, age-and sex-sensitive health interventions.

## Introduction

The Eastern Mediterranean Region (EMR) represents a mosaic of nations with marked differences in historical background, geopolitical and social context, fiscal capacity, cultural characteristics, and health care provision (1). According to the World Bank classification, this heterogeneity is also reflected in the region's internal stratification, with economies spanning from high-income to upper-middle-, lower-middle, and low-income levels (2).

In stating the EMR's epidemiological situation, communicable diseases (CDs) continue to be considered one of the most important public health problems. Each year, countries in the region experience multiple outbreaks of high-threat pathogens, exacerbated by fragile health systems and ongoing humanitarian crises (3). In 2021 alone, the region reported 31 major outbreaks of 11 epidemic-prone diseases, with over 398,000 cases and more than 680 related deaths, several of which carried the potential for global spread. Furthermore, the breakdown of health systems—driven by armed conflicts, large-scale population displacement, environmental disasters—has led to the resurgence of communicable diseases in EMR, often with devastating consequences (4). Protracted emergencies have further increased the risk of disease transmission due to overcrowded living conditions, inadequate sanitation, and limited access to healthcare (5).

Although most of the EMR countries have traditionally focused on combating infectious diseases, in recent years the burden of non-communicable diseases (NCDs)—such as cardiovascular diseases (CVDs), cancer, diabetes, and chronic respiratory diseases—seems to be rapidly increasing (6), accounting for a growing share of morbidity and mortality (7). According to the World Bank NCD Mortality Data, the EMR has one of the highest prevalence of NCDs globally, responsible for 79% of all deaths in the Middle East in 2019, exceeding the global average of 74% (8).

Many countries in the area, as well as globally, are now experiencing the simultaneous presence of communicable and non-communicable diseases, the so-called “double burden” of disease, as a natural phenomenon due to demographic and epidemiologic transitions (9). The convergence of NCDs and CDs has the potential to overstretch fragile health systems, particularly in low- and middle-income countries (LMICs). Health systems from these countries, originally structured to address acute infectious conditions, are now increasingly challenged by the need to manage chronic illnesses as well (10). This coexistence creates a unique challenge for health systems as they must address two sets of health priorities with different epidemiological profiles, prevention strategies, and treatment needs (8). The substantial implications are multiple, including overburdened health services, rising healthcare expenditures, and reduced economic productivity, all of which further hinder economic and human development (11).

In the EMR, a growing body of research highlights this complex challenge faced by national health systems (12). While many premature NCD deaths are preventable, limited health budgets, inadequate experience in managing chronic diseases, and a lack of political prioritization hinder progress in the region (13–15). A bibliometric analysis revealed a significant mismatch between the volume of research on cardiovascular diseases and their actual disease burden in the region (16). Additionally, much of the available data is based on estimates or forecasts—often derived from the Global Burden's Study (GBD)—rather than real-time surveillance, due to the absence of robust health surveillance systems. This limits the accuracy of health data for planning and policy development. As matter of facts, like all predictive models, these methods are subject to various uncertainties and limitations (17). Moreover, existing literature on the topic tends to focus on individual diseases within specific national contexts—for example, studies on NCDs in Gulf countries (18)—while few studies address the double burden of disease in an integrated perspective.

### The case of Iraq

Being part of the EMR, Iraq represents a relevant case study of the demographic and epidemiological transitions. During the 1970s, the country possessed one of the most advanced health systems in the Middle East (19). However, this system has progressively deteriorated over time, largely as a result of recurrent international and internal armed conflicts. Over the past 20 years, key health indicators have improved, with life expectancy at birth increasing from 69.5 years in 2000 to 71.5 years in 2021. The population, estimated at 45 million in 2023, is projected to increase by 60%, reaching ~72 million by 2050. Consequently, age-related non-communicable diseases are becoming increasingly prevalent. Currently, 19% of the population is under the age of 14, 76.7% are between 15 and 64, and 4.4% are aged 65 and older, indicating a young population that will increasingly require chronic care services in the future (20). Iraq is currently classified as a middle-income country.

According to the latest WHO EMR report, NCDs account for 54.7% of all deaths in the country (4), with a 22% risk of premature mortality from NCDs (21). Before 1980, the Iraqi health system was described as the best healthcare system in the Middle East. However, it has since experienced a severe decline in the availability of medical resources, funding, and healthcare personnel—mainly due to successive wars, international embargoes, economic sanctions, budget mismanagement, and widespread insecurity (22). The lack of a comprehensive nationwide system for health data collection and monitoring resulted in fragmented health information and poor-quality data that are neither timely nor reliable (23).

Specifically, in the Kurdistan Region of Iraq (KRI), little is known about the health status of the population, with only a few studies analyzing mortality trends in the area (24, 25). These studies indicate that CVDs are the leading cause of death, followed by neoplasms, infectious and parasitic diseases, and genitourinary diseases (26), with the burden of infectious and parasitic diseases remaining relatively stable over time. The increasing trend in the mean age at death and the growing share of deaths due to NCDs (27)—alongside persistently high rates of infectious diseases—mirror patterns observed across the broader Middle East (28).

### The health system in KRI

Since its establishment in the early 1990s, the Ministry of Health of the Kurdistan Regional Government has administered public sector health services, overseeing a broad network of primary and secondary care facilities. As in the rest of Iraq, the health system in the Kurdistan Region is organized on two levels, consisting of nearly sixty public hospitals and several hundred Primary Health Care Centers (PHCCs). PHCCs are classified into two types: main centers, located in urban and semi-urban areas, which provide a wide range of services including primary medical and dental care, immunizations, child growth monitoring, oral rehydration therapy for diarrheal disease, management of minor health conditions, and health education—and smaller centers, located in rural areas, which deliver a more limited package of services.

Despite their wide coverage, many PHCCs lack adequate infrastructure and equipment, such as laboratories, diagnostic tools, and information technologies. Main PHCCs are typically staffed with at least one general practitioner, supported by dentists, pharmacists, nurses, medical assistants, laboratory technicians, and administrative personnel. By contrast, sub-centers are usually staffed only with nurses and medical assistants and often do not have a physician.

### The health surveillance system in KRI

Until very recently, the Kurdistan Region had no health information system (HIS), and available health data were fragmented and largely based on indirect estimates or small-scale samples. Public health data—when collected—were mainly paper-based, with results presented only in aggregated form. Consequently, the format, accuracy, completeness, and accessibility of information represented major challenges in processing health statistics. In 2015, the University of Rome “Tor Vergata” and the Ministry of Health of the Kurdistan Region of Iraq, with financial support from the Italian Ministry of Foreign Affairs and the Italian Agency for Development Cooperation (AICS), launched the Kurdistan Regional Government District Health Information Software 2 (KRG-DHIS2) project, with the aim of establishing the first HIS in the region (27). The project's long-term goal is to integrate all centers in the region into the DHIS2 system. The project was designed to progressively collect health data by involving all public health facilities across the region. The implementation of the KRG-DHIS2 is still ongoing and being gradually expanded. The KRG-DHIS2 enables the progressive development of the first regional HIS, with the goal of transitioning from sample-based approaches to comprehensive population coverage (29). A reliable health information system is essential for modern policy development, as it underpins evidence-based decision-making, guides the delivery of healthcare assistance, and enables a timely response to health needs (30).

### Aim of the study

This study presents descriptive results from health data collected between 2016 and 2022 from facilities already integrated into the KRG-DHIS2, representing the initial stage of this ongoing process. Its main objective is to contribute to the limited body of evidence-based research by analyzing real-world data rather than estimates, thereby allowing an assessment of the burden of both communicable and non-communicable diseases in the KRI. To the best of our knowledge, this is the first study based on actual data derived from a digital health monitoring and epidemiological surveillance system, rather than on estimations or forecasts. The absence of reliable surveillance data has hindered the development of context-specific strategies for disease prevention and control; by addressing this gap, our findings aim to characterize the double burden of disease in Iraqi Kurdistan and provide actionable evidence to inform regional and national strategies for health system strengthening, resource allocation, and the prioritization of effective healthcare interventions.

## Materials and methods

### Inclusion of public health facilities in the KRG-DHIS2

As part of the main project, public health facilities (hospitals and primary health centers) were routinely included in the surveillance system according to specific selection criteria: serving a relatively large population, presence of medical staff, adequacy of technical infrastructure (including personnel and location), geographical distribution to ensure representativeness across all health directorates of the region, and willingness to participate. Each facility was visited prior to inclusion in the program to assess these characteristics.

### Routine data integration in the KRG-DHIS2

Health data collected at each participating center were assigned the appropriate diagnoses using the alphanumeric coding scheme (one letter followed by three numbers at the four-character level) for WHO International Statistical Classification of Diseases and Related Health Problems, Tenth Revision (ICD-10) (31). Diagnoses coded according to ICD-10 were recorded and transmitted to a central server on the DHIS2 platform. All data were fully anonymized, with no personal identifiers recorded, since they were collected solely for public health surveillance purposes. A full description of the workflow for developing and implementing the KRG-DHIS2 has been published previously (32).

### Training personnel

Staff from each participating public health facility—including medical doctors, nurses, and administrative personnel—received training on the DHIS2 platform, disease coding according to ICD-10, and data entry procedures. Training consisted of workshops and on-the-job sessions designed to ensure accurate and reliable data recording. To maintain data quality standards, refresher activities and continuous supervision, including remote follow-up, were implemented. In addition, a quality control system was established to routinely identify and correct implausible codes and incomplete records. This system combined automated checks with manual reviews conducted by trained supervisors.

### Database generation for the present analysis

The dataset analyzed in this study includes data from all public health centers that were progressively integrated into the KRG-DHIS2 up to 2022. The facilities considered were those that had been activated and had successfully started reporting during the study period. Inclusion was not based on predefined selection criteria but was determined by the centers' integration into the system since the project's inception. Therefore, the dataset should be considered a census of all included health centers rather than a sample.

To generate the study database, data were extracted from the KRG-DHIS2 platform and imported into Excel files. Diagnoses were initially grouped by ICD-10 category (one letter followed by two digits), then aggregated into blocks, and finally assigned to the corresponding ICD-10 chapters (based on the range of three-character categories).

According to the WHO classification (33) and the Global Burden Disease Study (GBD) (34), they were classified into four broad cause groups: Communicable, maternal, perinatal and nutritional conditions (CMNNDs), Non-communicable diseases (NCDs), Injuries and Ill-defined diseases (32–35).

CMNNDs encompass a broad set of conditions primarily caused by infectious agents and include major diseases of infectious etiology which remain among the leading global causes of morbidity and mortality. In addition, CMNNDs comprise all maternal and neonatal deaths, regardless of the immediate clinical cause, as these are largely preventable through effective public health interventions and access to essential healthcare services. Deaths resulting from nutritional deficiencies are also included, as they are closely linked to structural determinants such as poverty, food insecurity, and inadequate health coverage. Specifically, CMNNDs codification included the following ICD-10 chapter and single code: A00–B99, D50–D53, D64, E00–E02, E40–E46, E50–E64, G00–G04, G14, H65–H66, J00–J22, N70–N73, O00–O99, P00–P96, U04, U07, U09, and U10.

The broad category of NCDs includes a wide range of chronic conditions such as cardiovascular diseases, diabetes, cancers, chronic respiratory diseases, mental health disorders, and various other long-term health conditions often linked to modifiable behavioral risk factors. NCDs included the following ICD-10 chapter and single code: C00–C97, D00–D48, D55–D63, D65–D89, E03–E07, E10–E34, E65–E88, F01–F99, G06–G98-excluding G14, H00–H61, H68–H93, I00–I99, J30–J98, K00–K92, L00–L98, M00–M99, N00–N64, N75–N98, Q00–Q99, R95, U07, X41, X42, X44, and X45.

Injuries encompass a variety of external causes, including trauma-related injuries, drownings, poisonings, and animal or insect bites (ICD-10 codes: U12, V01–Y89 -excluding X41–X42 and X44–X45, S00–S99, and T00–T98).

Ill-defined diseases include a wide range of non-specific diagnoses, such as general symptoms and signs or abnormal findings from clinical and laboratory examinations, typically in cases where a precise diagnosis could not be established due to limited diagnostic information or incomplete clinical records (ICD-10 codes: R00–R94 and R96–R99). It should be noted that the classification into the four broad cause groups (CMNNDs, NCDs, Injuries, and Ill-defined diseases) was not always based on entire ICD-10 chapters. In some cases, only selected blocks or even specific single codes were included within a given category. Consequently, certain ICD-10 chapters are split across different groups. For instance, within the chapter Genitourinary System Diseases (N00–N99), codes N70–N73 were categorized under CMNNDs, while N00–N64 and N75–N98 were assigned to NCDs.

### Statistical analyses

Descriptive analyses are presented as absolute numbers and percentages for all categorical variables (sex, broad causes group, ICD-10 chapter, ICD-10 block), and as mean ± standard deviations (SD) and median for continuous variables (age). Data have been analyzed and presented for totals and split in CMNNDs and NCDs.

A LOESS (Locally Estimated Scatterplot Smoothing) (36, 37) curve was applied to highlight the age-related distribution of the five most frequent ICD-10 chapters, allowing for a flexible estimation of the trends in both CMNNDs and NCDs. LOESS was chosen because it is easy to interpret, widely used in descriptive epidemiology, and allows visualization of patterns without imposing parametric assumptions. All analyses were performed using R software (version 4.3.1). Given the purely descriptive nature of this study, no inferential or association analyses were performed.

## Results

**Figure 1:**
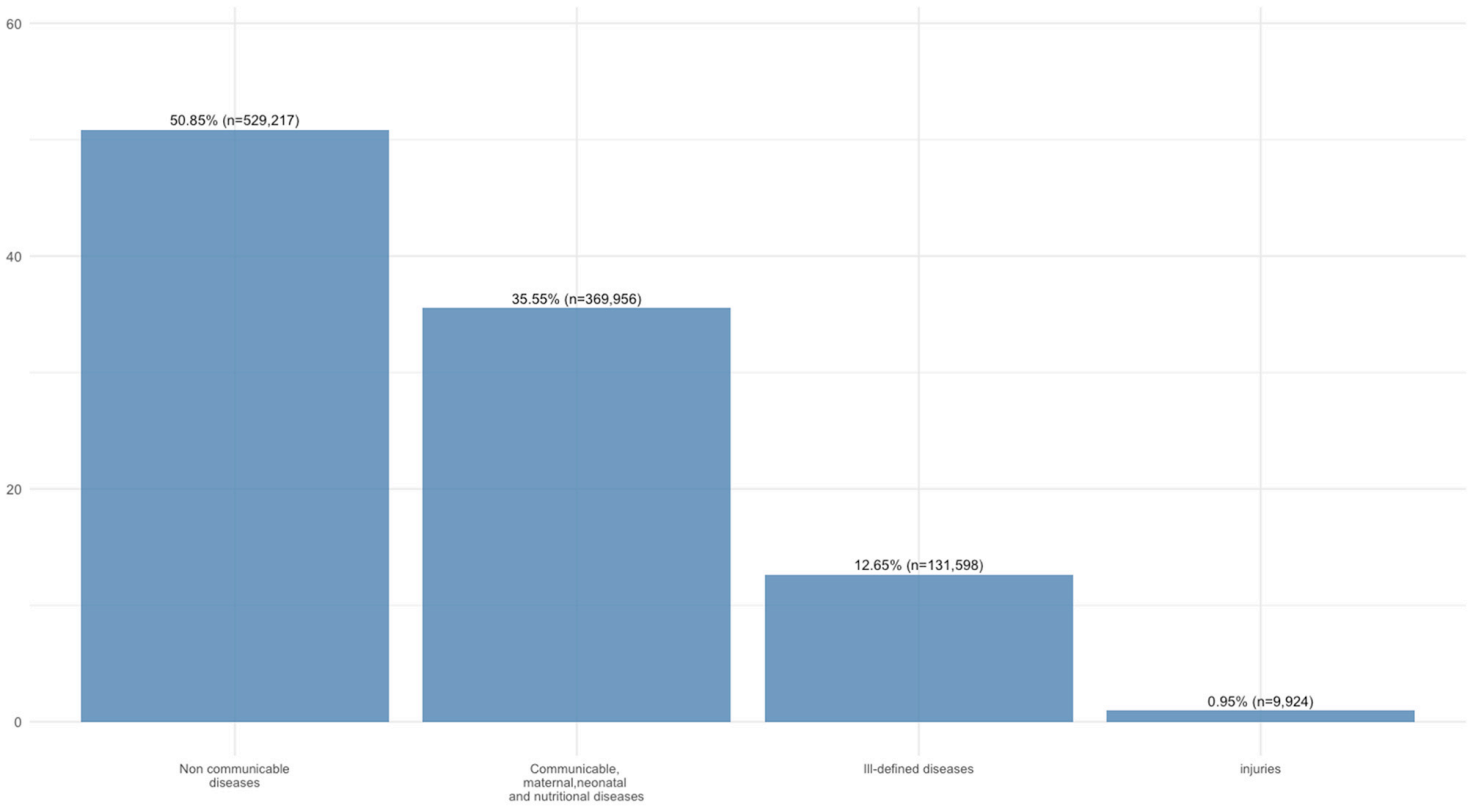
Diagnoses (both absolute numbers and percentages) by broad cause groups according to the WHO classification.

Between the years 2016 and 2022, 1,040,695 health events were recorded in the KRG-DHIS2 platform from 96 public health centers (PHC) in the population aged 18–90 years. Specifically, 54 facilities were located in the Duhok Directorate, 20 in Sulaimaniya, 17 in Erbil, and 5 in Halabja. With regard to location, 31 facilities were situated in rural areas and 65 in urban areas. These centers represent all the facilities that transmitted data to the health information system during the study period. Figure 1 presents health data according to the WHO broad causes classification: Communicable, maternal, perinatal and nutritional conditions (CMNNDs), Non-communicable diseases (NCDs), Injuries and Ill-defined diseases. The figure highlights the relative contribution of each category and clarifies the analytical sample used in subsequent analyses. Absolute numbers and percentages are reported above each bar. NCDs represented the largest group, accounting for slightly more than half of the sample (50.8%; *n* = 529,217). CMNNDs followed, contributing to over one third of the total health events (35.6%; *n* = 369,956). Ill-defined diseases (12.6%; *n* = 131,598) and Injuries (0.9%; *n* = 9,924) were less frequent, together contributing to <15% of the overall distribution. After excluding Ill-defined diseases and Injuries, the analytical sample consisted of 899,173 health events attributable to either CMNNDs or NCDs.

Table 1 shows descriptive data for the total sample and split by the two WHO groups' classification. Females constituted 63.4% of the total sample and showed a higher frequency of events within both broad cause groups. When considering CMNNDs and NCDs separately, the latter accounted for more events observed in both sexes. The overall mean age was 40.4 ± 14.6 years. Individuals with CMNN diseases were younger than those with NCDs, a pattern also reflected in the median age (36 vs. 40 years, respectively). As expected, the proportion of NCDs increased progressively with age, while an opposite trend was observed for CMNNDs. In every age group, NCDs accounted for a higher number of events compared to their CMNND counterparts, with the gradient becoming more pronounced in older age groups. Percentages reported in the table are calculated by row, indicating the distribution of CMNNDs and NCDs within each subgroup (sex and age category).

**Table 1:** Descriptive data for total sample and split by CMNNDs and NCDs.

**Variable**	**Total sample (*n* = 899,173)**	**CMNNDs (*n* = 369,956)**	**NCDs (*n* = 529,217)**
**Sex**, ***n*** **(%)**			
Males	328,881 (100)	146,278 (44.4)	182,603 (55.6)
Females	570,292 (100)	223,678 (39.2)	346,614 (60.8)
**Age**			
Mean ± SD	40.36 ± 14.6	37.9 ± 13.7	42.0 ± 15.0
Median	40.0	36.0	40.0
**Age group**, ***n*** **(%)**			
18-25 years	174,976 (100)	84,362 (48.2)	90,614 (51.8)
26–50 years	518,495 (100)	224,380 (43.3)	294,115 (56.7)
51–65 years	157,984 (100)	49,072 (31)	108,912 (68.9)
>65 years	47,718 (100)	12,142 (25.4)	35,576 (74.6)

In terms of overall cases, the most frequently diagnostic group were diseases of the respiratory system (J00–J99, *n* = 274,032; 30.5%), followed by genitourinary diseases (N00–N99, *n* = 152,565; 17.0%), digestive diseases (K00–K93, *n* = 126,224; 14.0%), musculoskeletal diseases (M00–M99, *n* = 76,779; 8.5%), and endocrine, nutritional, and metabolic diseases (E00–E90, *n* = 65,026; 7.2%). All other ICD-10 chapters each contributed <7% of overall cases (see Supplementary Table 1). Within respiratory diseases, the majority of cases were acute upper respiratory infections (64.8%), followed by other acute lower respiratory infections (18.1%), and influenza and pneumonia (15.3%). Genitourinary diseases were mainly due to urinary system conditions (73.8%), with smaller proportions of inflammatory (8.6%) and non-inflammatory (6.2%) female genital disorders. For digestive diseases, oral cavity, salivary gland, and jaw disorders accounted for two thirds of cases (67.1%), while intestinal diseases (16.5%) and upper gastrointestinal disorders (14.1%) were less frequent.

Females accounted for most cases across all ICD-10 chapters. The largest sex differences were observed for genitourinary system diseases (121,020 vs. 31,545 in males), endocrine and metabolic diseases (40,347 vs. 24,679), and blood diseases (15,258 vs. 3,006). Among males, digestive diseases were relatively more common than genitourinary diseases, but the overall ranking of the main chapters remained consistent.

Females were generally younger than their male counterparts across all disease categories. A notable difference of nearly 10 years was observed for blood diseases (median age: 33 years for females vs. 42 years for males) and for malignant neoplasms (median age: 50 vs. 60 years, respectively).

**Figure 2:**
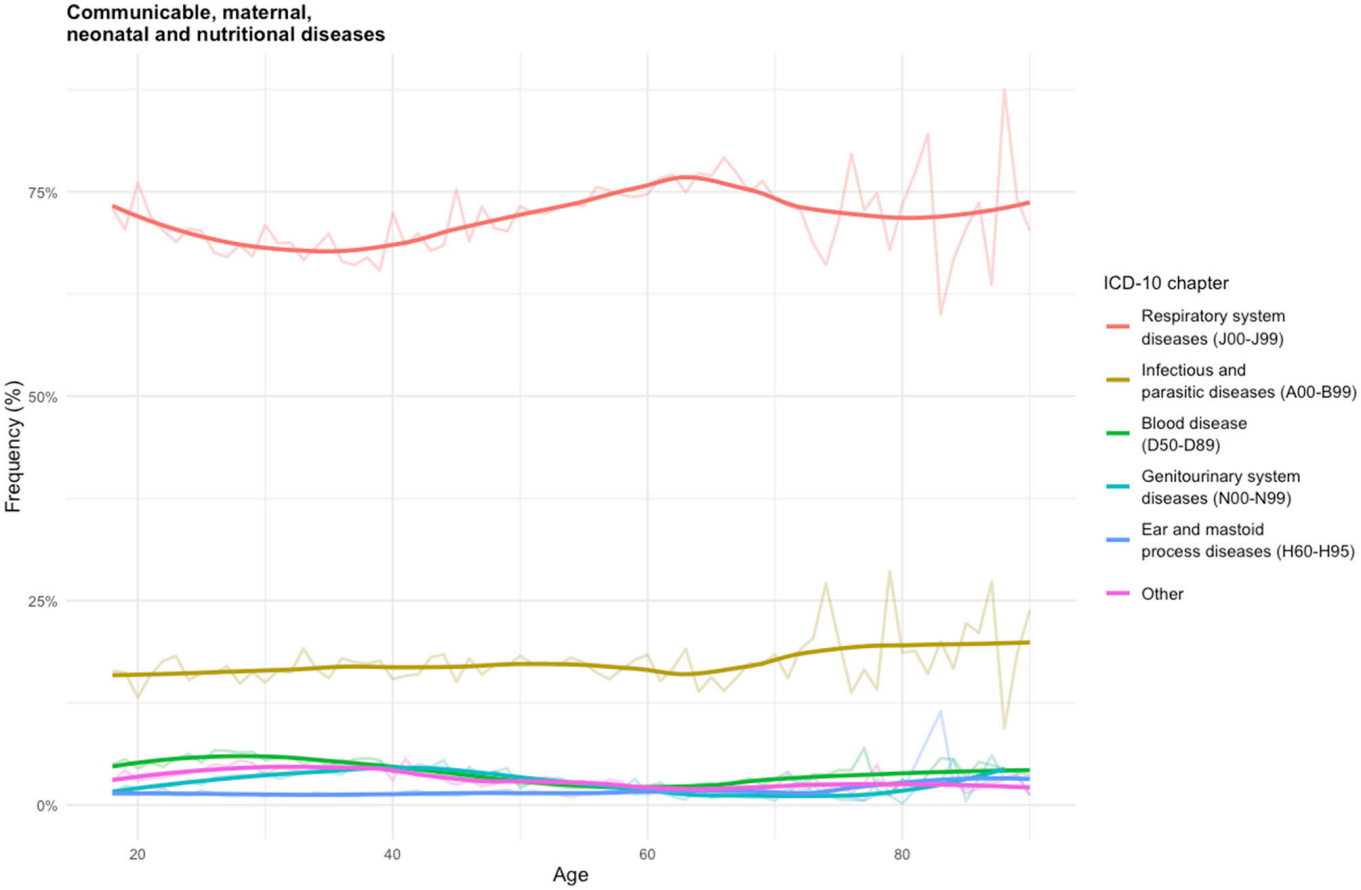
Age distribution of CMNNDs by ICD-10 chapters.

**Figure 3:**
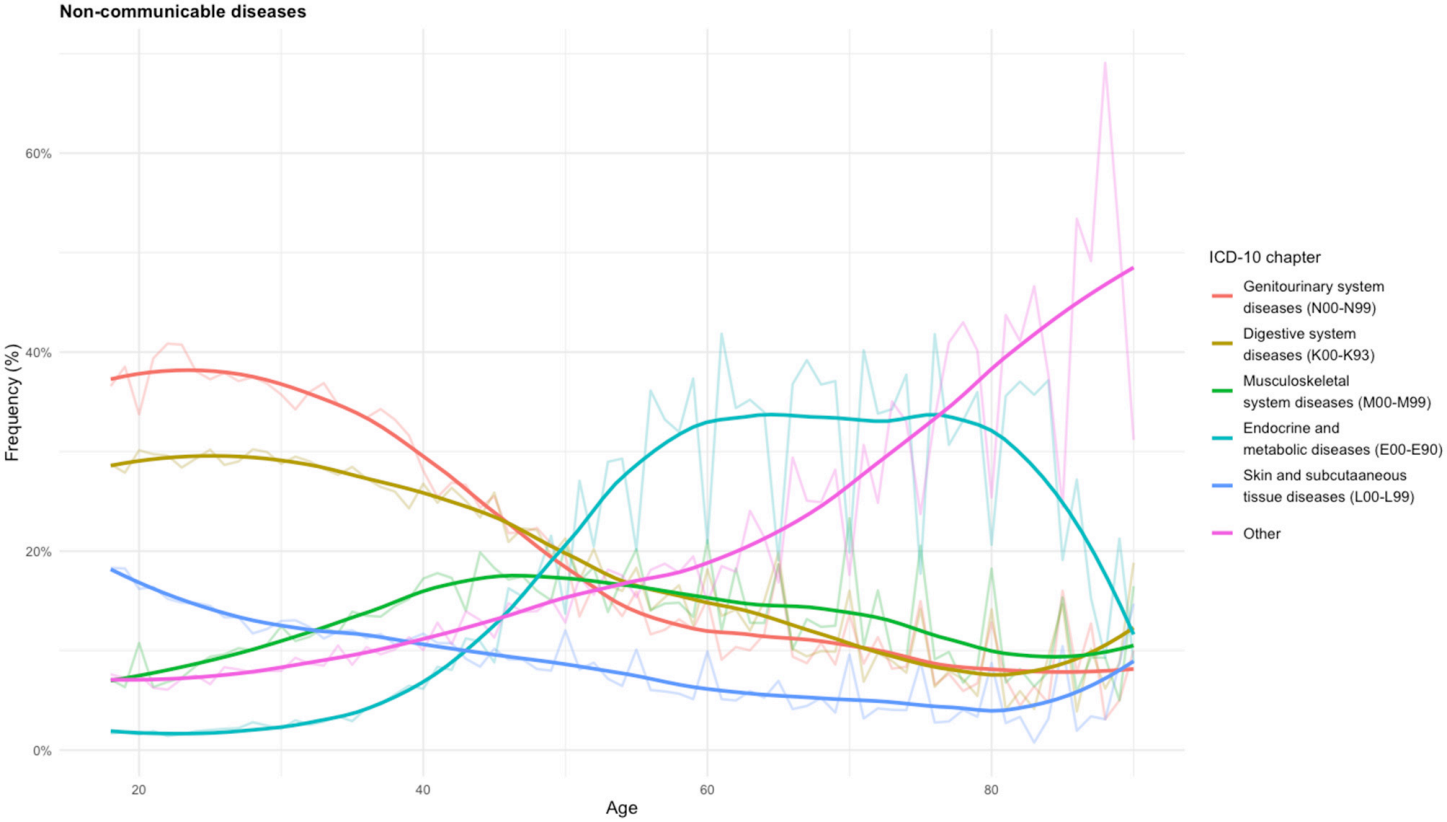
Age distribution of NCDs by ICD-10 chapters.

To better illustrate the distribution of events, Figure 2 and 3 present age-specific trends separately for CMNNDs and NCDs. For clarity, only the five most frequent diagnostic categories are shown, grouped according to ICD-10 chapters. The percentages on the y-axis represent the proportion of events within the corresponding category (CMNNDs or NCDs). Each curve shows the relative frequency of diagnoses within the relevant ICD-10 chapter according to patient age.

Figure 2 shows the age-specific frequency distribution of CMNNDs, expressed as the percentage of all CMNNDs-related events. Respiratory system diseases (J00–J99) represented the dominant category across all age groups, accounting for over 70% of cases, with a relatively stable trend across the age groups. Within this chapter, acute upper respiratory infections (J00-J06) alone represented about two thirds of all respiratory diagnoses (see Supplementary Table 2). Infectious and parasitic diseases (A00–B99) were the second most frequent category (around 16% of events), with a gradual increase among older adults, particularly after age 60. Over half of these events were attributable to intestinal infectious diseases (A00-A09), with similar frequencies between males and females. Other categories—including blood diseases (D50–D89), genitourinary system diseases (N00–N99), ear and mastoid process diseases (H60–H95), and remaining ICD-10 chapters—each accounted for <5% of cases. These latter categories display a modest decline in frequency between ages 30 and 60, followed by a relatively stable pattern in both younger and older age groups, with the exception for ear and mastoid process diseases, which continued to be more prevalent in children. Notable sex differences were observed in blood diseases (*n* = 14,075 vs. *n* = 2,268) and genitourinary system diseases (*n* = 11,233 vs. *n* = 175), both more frequent in females. No consistent age-related differences between sexes were observed across CMNND categories.

Figure 3 displays the age-specific frequency distribution of selected NCDs, expressed as a percentage of all NCD-related events. The most frequent categories were genitourinary system diseases (N00–N99, >26%) and digestive system diseases (K00–K93, <23%) (see Supplementary Table 3). Both conditions were more prevalent in younger ages and progressively declined with age. Genitourinary diseases were more frequent among females (*n* = 109,787 vs. *n* = 31,370) with male cases occurring at an older median age (about 5 years differences). Within this chapter, other diseases of the urinary system (N30-N39) were predominant. In contrast, digestive system diseases represented the most frequently reported NCD category among males, accounting for 27.7% of their diagnoses. Musculoskeletal diseases (M00–M99) represented about 10% of cases and exhibited a clear age-related pattern: rising steadily from early adulthood, peaking between ages 40 and 60, and then slightly declining, though remaining higher than in youth. Arthropathies (M00-M25) were the main represented blocks (67.3%). Endocrine and metabolic diseases (E00–E90) showed a bimodal distribution, with higher frequencies observed between 60 and 70, followed by a sharp decline. Over 90% of these cases were due to diabetes mellitus. Skin and subcutaneous tissue diseases (L00–L99) represented nearly 20% of cases across all age groups and showed a decreasing trend with age, although less pronounced. They were proportionally more frequent among males. Dermatitis and eczema (L20–L30) were the main subgroups. Other ICD-10 chapters exhibited a marked and continuous increase with age.

## Discussion

The global population is increasingly facing a dual burden of communicable and non-communicable diseases, though the magnitude and stage of this burden vary significantly across countries. Many countries—particularly low- and middle-income nations and fragile settings affected by instability, prolonged crises, and conflicts—continue to rely on health estimates that often lack precision, representativeness, and comprehensive coverage. In such contexts, systematically collected surveillance data and accurate information on disease patterns and healthcare utilization are essential to strengthen health systems with greater reliability and contextual relevance. These efforts are closely tied to the quality of universal health coverage (UHC) and the performance of primary health care systems (38). Of the six building blocks of a health system, timely and reliable health surveillance is essential for making informed public health decisions, designing effective interventions, and allocating resources appropriately (39). It is essential to address the rising burden of NCDs and the persistent threat posed by emerging and re-emerging communicable diseases to advance UHC in the Eastern Mediterranean Region (EMR) (3, 5). As the WHO has emphasized, “There is no health without research.” Evidence-based research is therefore crucial to generating high-quality data that can inform policy, clinical practice, and investment in health systems, particularly in LMICs (40).

As highlighted by the WHO, official mortality data from Iraq are considered highly unreliable. The WHO country database provides mortality statistics only, as morbidity and diagnosis-related data remain unavailable due to the greater challenges of systematic collection. In contrast to most previous studies in Iraq, which have relied primarily on estimates and forecasting models rather than systematically collected information, our analysis draws on real-time, routinely recorded clinical data from public health facilities using ICD-10 coding. To the best of our knowledge, this is the first study of its kind in the Iraqi Kurdistan—and possibly in the entire country—to systematically analyze facility-based data from individuals accessing public healthcare services through a digital health monitoring system. We conducted a descriptive analysis to present and summarize the overall health data collected thus far from the public health facilities activated within the KRG-DHIS2. Drawing on 8 years of surveillance data collected through this digital public health platform, the present study provides insights into the combined impact of CMNNDs and NCDs. These data describe the disease burden at the population level and should not be interpreted as reflecting the clinical condition of an individual.

Our results highlight the double burden of disease, with NCDs accounting for over half of all health records and CMNNDs accounting for over one-third. Our analysis revealed distinct age-related trends in disease distribution, which underscores the importance of age-specific prevention and management strategies. As expected, CMNNDs were more prevalent among younger age groups, while NCDs increased with age (10). These findings reflect the ongoing demographic and epidemiological transition and support the development of age-specific screening and health promotion programs. In the case of NCDs, multiple determinants contribute to their rising prevalence in Iraq, including shifts in dietary habits toward energy-dense foods, declining levels of physical activity, and high rates of tobacco use (41). Decades of conflict and instability have further undermined the health system, limiting both preventive and curative services (42). As a result, the Iraqi healthcare system faces growing challenges in meeting the demands posed by the increasing NCD burden (43).

One of the key findings of this analysis is the high volume of visits to public primary healthcare facilities for NCDs, particularly diabetes and cancer (18). These diseases usually necessitate long-term management and specialized care, which suggests an absence or limited accessibility of referral services in the region. Reliance on primary care for these diagnoses likely reflects an absence of adequate specialist infrastructure (44). Although diabetes is currently reported to be low in Iraq, it is expected to increase, placing a growing burden on the health system. This trend mirrors those observed in other parts of the Middle East (21).

The cancer burden is particularly concerning given the deterioration of cancer care in Iraq over the past three decades, which has caused services to fall below international standards. Our data show that breast cancer accounted for over 16% of all malignant neoplasm records, confirming its status as one of the country's most prevalent cancers, as reported in previous investigations (45).

Additionally, respiratory diseases were the most commonly diagnosed conditions across all age groups (5), with acute upper respiratory infections accounting for over 65% of cases. The high proportion may reflect a combination of factors, including seasonal epidemics of respiratory pathogens such as seasonal influenza (46). Since chronic respiratory conditions were less frequently recorded, this may reflect limitations in diagnosis and reporting, as well as broader regional challenges in accurately assessing the burden of chronic respiratory diseases. Indeed, healthcare services and data for these conditions remain underdeveloped in many Middle Eastern countries (47). Environmental exposures further contribute to this pattern. In EMR, populations are highly exposed to dust storms, which represent a major source of air pollution. At the same time, anthropogenic emissions—particularly from traffic, industrial activity, and the use of biomass fuels for household energy—have become a leading health risk (48, 49). Recent epidemiological evidence has demonstrated that air pollution increases the risk of both morbidity and mortality from respiratory and cardiovascular diseases in the region (50). Advancing and utilizing more sound epidemiological designs and studies on the effect of air pollution on the respiratory health outcomes is needed to portray the actual situation EMR (51).

Sex-based differences were also observed. Women accounted for a larger proportion of health events and had higher rates of visits for endocrine, genitourinary, and hematologic disorders. These patterns align with previous studies attributing higher healthcare utilization among women to reproductive health needs and stronger health-seeking behaviors (52). While biological predispositions may partly explain these patterns, sociocultural and health system factors appear equally important. These differences likely reflect disparities in healthcare access and service utilization rather than true variations in disease prevalence, which can only be accurately assessed at the population level. Women's more frequent contact with healthcare services through reproductive and maternal health programs facilitates higher detection rates, whereas cultural barriers and stigma surrounding genitourinary conditions in men may contribute to underreporting and delayed diagnosis. Differences in access to specialized services and diagnostic capacities across facilities may further shape these trends. The relatively lower utilization of services by men highlights the need for targeted outreach strategies to enhance male engagement with healthcare.

It is difficult to compare our findings with other studies conducted in Iraq, as most available research has focused on causes of mortality rather than on the broader spectrum of diseases leading individuals to seek healthcare. Nonetheless, it is worth noting that, while substantial progress has been achieved in reducing mortality among younger populations—particularly for chronic respiratory diseases—persistent high mortality rates among older males, especially from cardiovascular conditions, highlight the need for more targeted interventions (53).

These findings require immediate attention from public health policymakers, especially regarding resource allocation for disease identification, prevention, and treatment. Therefore, the availability of an integrated nationwide disease surveillance system is essential (41, 42).

### Limitations

This study has several limitations that should be acknowledged. First, individuals self-reported their age. As a result, there may be minor inaccuracies, which could affect the precision of age-specific analyses. Second, it was not possible to link multiple healthcare visits to individual patients because the dataset did not include unique identifiers. This limitation may underestimate the burden of chronic conditions because individuals with long-term illnesses are likely to seek healthcare more frequently, a pattern that could not be assessed in this analysis. Third, diagnoses were recorded only for the primary reason for the healthcare visit. Therefore, the co-occurrence of multiple conditions in the same individual could not be evaluated, and the dataset may not fully reflect the complexity of patients' health status, including comorbidities. For this reason, the concept of the double burden is applied at the population level rather than at the individual level. Future developments of the health information system may allow for analyses that capture the overlap of conditions within individuals. Fourth, health centers contributing data were not evenly distributed across the Kurdistan Region of Iraq. These facilities were not selected based on predefined sampling strategies but rather corresponded to those progressively activated within the DHIS2 system during the study period. This may limit representativeness, and future research should extend data collection to include a wider range of healthcare facilities and geographic areas—ideally covering the entire country—to enhance generalizability and support more comprehensive public health planning. Moreover, the estimation of longitudinal trends was not feasible in this analysis. Although 8 years of data were available, health centers were activated progressively rather than simultaneously, which reduces the comparability of time-series analyses. Finally, despite targeted training of healthcare personnel in ICD-10 coding and data entry, and the implementation of several quality control measures, errors in diagnosis or coding cannot be ruled out. Such inaccuracies are an inherent limitation of real-world health information systems and cannot be entirely eliminated.

### Strengths of the study

This study provides critical evidence for understanding health service utilization and disease burden in KRI, where health data systems are underdeveloped. This unique dataset provides a reliable, granular, contextualized understanding of population health needs, offering a solid foundation for informed public health planning and resource allocation. Furthermore, the study highlights the potential of facility-based digital data collection to inform priority setting and health system strengthening in post-conflict or transitional contexts. Finally, this work raises important questions about why adults seek care in public facilities. Understanding the factors that influence health facility attendance, such as access barriers, perceptions of care quality, medication availability, and referral options, is essential to improving service delivery and health outcomes in the region. Digital health technologies, including electronic health records and real-time monitoring systems, have the potential to transform regions like KRI, where health systems face constraints. The implementation of ICD-10 coding in this context represents a methodological strength, as it ensures international comparability and standardization of disease classification, enhances interoperability with global datasets, and facilitates evidence-based decision-making at both local and national levels. Drawing on real-world data from public health facilities, our findings underscore the urgent need to strengthen digital surveillance systems, expand access to integrated healthcare services, and adapt public health interventions to evolving health needs. In particular, they identify priority areas for resource allocation, the consolidation and expansion of surveillance infrastructures such as the KRG-DHIS2, and the integration of communicable and non-communicable disease programs through coordinated prevention, early detection, and treatment strategies. Strengthening non-communicable disease prevention and screening—especially among older adults—and more systematically integrating chronic disease management into primary health care should be considered essential priorities for the future. Addressing these domains can support the design of context-specific policies that enhance health system resilience, improve population health outcomes, and guide long-term investments in sustainable healthcare delivery. Moreover, digital health tools can improve the quality, efficiency, and responsiveness of care, even during acute emergencies that threaten to overwhelm existing infrastructure. In parallel, leveraging big data analytics offers the potential to strengthen early detection and outbreak control, provided that adequate investments are made in both technical capacity and human resources (43).

In conclusion, this study offers a comprehensive overview of the double burden of disease in the Iraqi Kurdistan, a region undergoing demographic and epidemiological transitions and engaged in post-crisis rebuilding. National health policies, strategies, and plans must respond to these emerging epidemiological realities in Iraq, positioning it as a best practice model for the broader Middle East.

## Data Availability

The raw data supporting the conclusions of this article will be made available by the authors, without undue reservation.
