# Supplementary material for: Exploring health needs and the double burden of disease in adults attending public health facilities in the Iraqi Kurdistan
Source: Front Public Health. 2025 Oct 23;13:1649273. doi: 10.3389/fpubh.2025.1649273 (PMC12589055; doi:10.3389/fpubh.2025.1649273)
Supplement: Supplementary file 1 [file Table_1.docx]

**Supplementary Table 1. Top 3 blocks by 10 main ICD-10 chapters for total events.**

| **TOTAL SAMPLE** | **Top 10 chapters with 3 main blocks** | **N. of events** | **%** | **Sex**  **N. (%)** | **Median age** | **Mean age ±SD** |
| --- | --- | --- | --- | --- | --- | --- |
| **ICD-10 chapter**  **J00-J99** | **Respiratory system diseases** | **274,032** | **30.5** | **F 153,532 (26.9)**  **M 120,500 (36.6)** | **F 36.0**  **M 37.0** | **F 37.9 ± 13.5**  **M 38.8 ±14.4** |
| ICD-10 block  J00-J06 | Acute upper respiratory infections | 177,622 | 64.8 |  |  |  |
| ICD-10 block  J20-J39 | Other acute lower respiratory infections | 49,582 | 18.1 |  |  |  |
| ICD-10 block  J09-J18 | Influenza and pneumonia | 41,871 | 15.3 |  |  |  |
|  | Others | 4,957 | 1.8 |  |  |  |
| **ICD-10 chapter**  **N00-N99** | **Genitourinary system diseases** | **152,565** | **17.0** | **F 121,020 (21.2)**  **M 31,545 (9.5)** | **F 35.0**  **M 39.0** | **F 35.9 ± 12.3**  **M 40.2 ±15.1** |
| ICD-10 block  N30-N39 | Other urinary system diseases | 112,608 | 73.8 |  |  |  |
| ICD-10 block  N70-N77 | Inflammatory female pelvic organ diseases | 13,095 | 8.6 |  |  |  |
| ICD-10 block  N80-N98 | Non-inflammatory female genital tract disorders | 9,450 | 6.2 |  |  |  |
|  | Others | 17,412 | 11.4 |  |  |  |
| **ICD-10 chapter**  **K00-K93** | **Digestive system diseases** | **126,224** | **14.0** | **F 75,601 (13.2)**  **M 50,623 (15.3)** | **F 37.0**  **M 38.0** | **F 38.1 ± 13.2**  **M 39.4 ±14.4** |
| ICD-10 block  K00-K14 | Oral cavity, salivary glands and jaws diseases | 84,710 | 67.1 |  |  |  |
| ICD-10 block  K55-K64 | Other intestinal diseases | 20,786 | 16.5 |  |  |  |
| ICD-10 block  K20-K31 | Oesophagus, stomach and duodenum diseases | 17,771 | 14.1 |  |  |  |
|  | Others | 2,957 | 2.3 |  |  |  |
| **ICD-10 chapter M00-M99** | **Musculoskeletal system diseases** | **76,779** | **8.5** | **F 50,095 (8.7)**  **M 26,684 (8.1)** | **F 45.0**  **M 45.0** | **F 45.0 ± 13.6**  **M 45.5 ±14.5** |
| ICD-10 block  M00-M25 | Arthropathies | 51,679 | 67.3 |  |  |  |
| ICD-10 block  M40-M54 | Dorsopathies | 13,389 | 17.4 |  |  |  |
| ICD-10 block  M60-M79 | Soft tissue disorders | 10,624 | 13.8 |  |  |  |
|  | Others | 1,087 | 1.5 |  |  |  |
| **ICD-10 chapter**  **E00-E90** | **Endocrine and Metabolic diseases** | **65,026** | **7.2** | **F 40,347 (7.0)**  **M 24,679 (7.5)** | **F 54.0**  **M 56.0** | **F 52.6 ± 13.0**  **M 55.1 ±12.0** |
| ICD-10 block  E10-E14 | Diabetes mellitus | 56,824 | 87.4 |  |  |  |
| ICD-10 block  E50-E64 | Other nutritional deficiencies | 3,258 | 5.0 |  |  |  |
| ICD-10 block  E00-E07 | Thyroid gland disorders | 2,778 | 4.3 |  |  |  |
|  | Others | 2,166 | 3.3 |  |  |  |
| **ICD-10 chapter**  **A00-B99** | **Infectious diseases** | **60,837** | **6.8** | **F 37,457 (6.5)**  **M 23,380 (7.1)** | **F 36.0**  **M 38.0** | **F 37.7 ± 13.3**  **M 39.5 ±14.5** |
| ICD-10 block  A00-A09 | Intestinal infectious diseases | 31,389 | 51.6 |  |  |  |
| ICD-10 block  B35-B49 | Mycoses | 13,365 | 22.0 |  |  |  |
| ICD-10 block  B00-B09 | Viral infections by skin and mucous membrane lesions | 6,228 | 10.2 |  |  |  |
|  | Others | 9,855 | 16.2 |  |  |  |
| **ICD-10 chapter**  **L00-L99** | **Skin and subcutaneous diseases** | **58,410** | **6.5** | **F 35,514 (6.2)**  **M 22,896 (6.9)** | **F 35.0**  **M 39.0** | **F 37.0 ± 13.5**  **M 39.9 ±15.2** |
| ICD-10 block  L20-L30 | Dermatitis and eczema | 36,518 | 62.5 |  |  |  |
| ICD-10 block  L60-L75 | Skin appendages disorders | 7,166 | 12.3 |  |  |  |
| ICD-10 block  L00-L08 | Skin and subcutaneous tissue infections | 5,356 | 9.2 |  |  |  |
|  | Others | 9,370 | 16.0 |  |  |  |
| **ICD-10 chapter**  **I00-I99** | **Circolatory system diseases** | **25,657** | **2.8** | **F 14,955 (2.6)**  **M 10,702 (3.2)** | **F 52.0**  **M 55.0** | **F 52.4 ± 13.3**  **M 54.7 ±12.9** |
| ICD-10 block  I10-I15 | Hypertensive diseases | 17,760 | 69.2 |  |  |  |
| ICD-10 block  I30-I52 | Other forms of hearth diseases | 5,674 | 22.1 |  |  |  |
| ICD-10 block  I95-I99 | Other and unspecified circulatory system disorders | 1,743 | 6.9 |  |  |  |
|  | Others | 480 | 1.8 |  |  |  |
| **ICD-10 chapter**  **D50-D89** | **Blood diseases** | **18,264** | **2.0** | **F 15,258 (2.6)**  **M 3,006 (0.9)** | **F 33.0**  **M 42.0** | **F 34.7 ± 11.8**  **M 42.9 ±16.7** |
| ICD-10 block  D60-D64 | Aplastic and other anaemias | 8,898 | 48.7 |  |  |  |
| ICD-10 block  D50-D53 | Nutritional anaemias | 7,597 | 41.6 |  |  |  |
| ICD-10 block  D65-D69 | Coagulation defects, purpura and other haemorrhagic conditions | 1,558 | 8.5 |  |  |  |
|  | Others | 211 | 1.2 |  |  |  |
| **ICD-10 chapter**  **C00-D48** | **Malignant neoplasms** | **10,126** | **1.1** | **F 5,698 (1.0)**  **M 4,428 (1.3)** | **F 50.0**  **M 60.0** | **F 50.3 ± 15.8**  **M 57.5 ±17.1** |
| ICD-10 block  C15-C26 | Digestive organs malignant neoplasms | 2,224 | 22.0 |  |  |  |
| ICD-10 block  C50 | Breast malignant neoplasm | 1,711 | 16.9 |  |  |  |
| ICD-10 block  C81-C96 | Lymphoid, haematopoietic and related tissue malignant neoplasms | 1,394 | 13.8 |  |  |  |
|  | Others | 4,797 | 47.3 |  |  |  |
| **Others** |  | **31,253** | **3.6** |  |  |  |
