# Supplementary material for: Exploring health needs and the double burden of disease in adults attending public health facilities in the Iraqi Kurdistan
Source: Front Public Health. 2025 Oct 23;13:1649273. doi: 10.3389/fpubh.2025.1649273 (PMC12589055; doi:10.3389/fpubh.2025.1649273)
Supplement: Supplementary file 2 [file Table_2.docx]

**Supplementary Table 2. Top 3 blocks by 5 main ICD-10 chapters for CMNND events.**

| **CMNNDs** | **Top 5 chapters with 3 main blocks** | **N. of events** | **%** | **Sex**  **N. (%)** | **Median age** | **Mean age ±SD** |
| --- | --- | --- | --- | --- | --- | --- |
| **ICD-10 chapter**  **J00-J99** | **Respiratory system diseases** | **263,267** | **71.2** | **F 147,569 (65.9)**  **M 115,698 (79.1)** | **F 36.0**  **M 37.0** | **F 37.8 ±13.4**  **M 38.7±14.4** |
| ICD-10 block  J00-J06 | Acute upper respiratory infections | 177,622 | 67.6 |  |  |  |
| ICD-10 block  J20-J39 | Other acute lower respiratory infections | 43,774 | 16.6 |  |  |  |
| ICD-10 block  J09-J18 | Influenza and pneumonia | 41,871 | 15.9 |  |  |  |
|  | Others | 0 | 0.0 |  |  |  |
| **ICD-10 chapter A00-B99** | **Infectious diseases** | **60,837** | **16.4** | **F 37,457 (16.7)**  **M 23,380 (16.0)** | **F 36.0**  **M 38.0** | **F 37.7 ± 13.3**  **M 39.5±14.5** |
| ICD-10 block  A00-A09 | Intestinal infectious diseases | 31,389 | 51.6 |  |  |  |
| ICD-10 block  B35-B49 | Mycoses | 13,365 | 22.0 |  |  |  |
| ICD-10 block  B00-B09 | Viral infections by skin and mucous membrane lesions | 6,228 | 10.2 |  |  |  |
|  | Others | 9,855 | 16.2 |  |  |  |
| **ICD-10 chapter D50-D89** | **Blood diseases** | **16,343** | **4.4** | **F 14,075 (6.3)**  **M 2,268 (1.5)** | **F 32.0**  **M 36.0** | **F 34.0 ± 11.4**  **M 39.0±16.1** |
| ICD-10 block  D60-D64 | Aplastic and other anaemias | 8,746 | 53,5 |  |  |  |
| ICD-10 block  D50-D53 | Nutritional anaemias | 7,597 | 46.5 |  |  |  |
|  | Others | 0 | 0.0 |  |  |  |
| **ICD-10 chapter N00-N99** | **Genitourinary system diseases** | **11,408** | **3.1** | **F 11,233 (5.0)**  **M 175 (0.1)** | **F 36.0**  **M 35.0** | **F 36.9 ± 10.7**  **M 37.0±11.4** |
| ICD-10 block  N70-N77 | Inflammatory female pelvic organs diseases | 11,408 | 100.0 |  |  |  |
|  | Others | 0 | 0.0 |  |  |  |
| **ICD-10 chapter H60-H95** | **Ear and mastoid process diseases** | **5,015** | **1.4** | **F 2,730 (1.2)**  **M 2,285 (1.5)** | **F 36.0**  **M 37.0** | **F 38.0 ± 13.5**  **M 38.8±15.1** |
| ICD-10 block  H65-H75 | Middle ear and mastoid diseases | 5,015 | 100.0 |  |  |  |
|  | Others | 0 | 0.0 |  |  |  |
| **Others** |  | **13,086** | **3.5** |  |  |  |
