# Supplementary material for: Exploring health needs and the double burden of disease in adults attending public health facilities in the Iraqi Kurdistan
Source: Front Public Health. 2025 Oct 23;13:1649273. doi: 10.3389/fpubh.2025.1649273 (PMC12589055; doi:10.3389/fpubh.2025.1649273)
Supplement: Supplementary file 3 [file Table_3.docx]

**Supplementary Table 3. Top 3 blocks by 5 main ICD-10 chapters for NCD events.**

| **NCDs** | **Top 5 chapters with 3 main blocks** | **N. of events** | **%** | **Sex**  **N. (%)** | **Median age** | **Mean age ±SD** |
| --- | --- | --- | --- | --- | --- | --- |
| **ICD-10 chapter**  **N00-N99** | **Genitourinary system diseases** | **141,157** | **26.7** | **F 109,787 (31.6)**  **M 31,370 (17.1)** | **F 35.0**  **M 39.0** | **F 35.8±12.5**  **M 40.3±15.2** |
| ICD-10 block  N30-N39 | Other urinary system diseases | 112,608 | 79.8 |  |  |  |
| ICD-10 block  N80-N98 | Noninflammatory female genital tract disorders | 9,450 | 6.7 |  |  |  |
| ICD-10 block  N20-N23 | Urolithiasis | 7,559 | 5.4 |  |  |  |
|  | Others | 11,540 | 8.1 |  |  |  |
| **ICD-10 chapter**  **K00-K93** | **Digestive system diseases** | **126,224** | **23.9** | **F 75,601 (21.8)**  **M 50,623 (27.7)** | **F 37.0**  **M 38.0** | **F 38.1 ± 13.2**  **M 39.4 ± 14.5** |
| ICD-10 block  K00-K14 | Oral cavity, salivary glands and jaws diseases | 84,710 | 67.1 |  |  |  |
| ICD-10 block  K55-K64 | Other intestinal diseases | 20,786 | 16.5 |  |  |  |
| ICD-10 block  K20-K31 | Oesophagus, stomach and duodenum diseases | 17,771 | 14.1 |  |  |  |
|  | Others | 2,957 | 2.3 |  |  |  |
| **ICD-10 chapter**  **M00-M99** | **Musculoskeletal system diseases** | **76,779** | **14.5** | **F 50,095 (14.4)**  **M 26,684 (14.6)** | **F 45.0**  **M 45.0** | **F 45.0 ± 13.6**  **M 45.5 ± 14.5** |
| ICD-10 block  M00-M25 | Arthropathies | 51,679 | 67,3 |  |  |  |
| ICD-10 block  M40-M54 | Dorsopathies | 13,389 | 17.4 |  |  |  |
| ICD-10 block  M60-M79 | Soft tissue disorders | 10,624 | 13.8 |  |  |  |
|  | Others | 1,087 | 1.2 |  |  |  |
| **ICD-10 chapter**  **E00-E90** | **Endocrine and Metabolic diseases** | **61,414** | **11.6** | **F 37,606 (10.8)**  **M 23,808 (13.0)** | **F 55.0**  **M 56.0** | **F 53.7 ± 12.5**  **M 55.5 11.7** |
| ICD-10 block  E10-E14 | Diabetes mellitus | 56,824 | 92.5 |  |  |  |
| ICD-10 block  E00-E07 | Thyroid gland disorders | 2,760 | 4.5 |  |  |  |
| ICD-10 block  E20-E35 | Other endocrine glands disorders | 898 | 1.5 |  |  |  |
|  | Others | 898 | 1.5 |  |  |  |
| **ICD-10 chapter**  **L00-L99** | **Skin and subcutaneous diseases** | **58,410** | **11.0** | **F 35,514 (10.2)**  **M 22,896 (12.5)** | **F 35.0**  **M 39.0** | **F 37.1 ± 13.5**  **M 39.9 ± 15.3** |
| ICD-10 block  L20-L30 | Dermatitis and eczema | 36,518 | 62.5 |  |  |  |
| ICD-10 block  L60-L75 | Skin appendages disorders | 7,166 | 12.3 |  |  |  |
| ICD-10 block  L00-L08 | Skin and subcutaneous tissue infections | 5,356 | 9.2 |  |  |  |
|  | Others | 9,370 | 16.0 |  |  |  |
| **Others** |  | **65,233** | **12.3** |  |  |  |
